# Effect of different growing media on pomological and phytochemical parameters of *Fragaria vesca* ʻYellow Wonderʼ and *Fragaria ×ananassa* ʻCamarosaʼ: a comparative study

**DOI:** 10.1186/s12870-023-04409-2

**Published:** 2023-08-23

**Authors:** Nafiye Unal, Ibrahim Kahramanoglu, Hanifeh Seyed Hajizadeh, Jale Bilgin, Volkan Okatan

**Affiliations:** 1 Department of Horticulture, Faculty of Agriculture, Akdeniz University, Antalya, Turkey; 2 Department of Horticulture, Faculty of Agricultural Sciences and Technologies, European University of Lefke, Gemikonagi, Mersin, 99780 Northern Cyprus Turkey; 3 Department of Horticulture, Faculty of Agriculture, University of Maragheh, Maragheh, 55136-553 Iran; 4 Department of Horticulture, Faculty of Agriculture, Eskişehir Osmangazi University, Eskişehir, 26160 Turkey

**Keywords:** Soil, Soilless, *Fragaria vesca*, *Fragaria ****×**** ananassa*, Phytochemical, Comparative

## Abstract

**Background:**

Strawberries are one of the most widely cultivated fruits in the world, and their popularity continues to grow due to their unique taste, high nutritional value, and numerous health benefits. The success of strawberry cultivation depends largely on the quality of the growing media used. In recent years, there has been a growing interest in soilless media as a sustainable alternative to traditional soil-based growing methods. This study aimed to compare the effect of different growing media, both soil and soilless (Hydroponic Production System) media, on the fruit quality and phytochemical contents of two cultivars of strawberry (Yellow Wonder and Camarosa) in a greenhouse.

**Results:**

The values of Fruit weight, fruit firmness, and SSC were higher in soilless media than in soil media. In addition, ʻCamarosaʼ was higher than ʻYellow Wonderʼ in these characteristics. The rates of glucose and fructose were higher in soil media than soilless media, and ʻYellow Wonderʼ was higher than ʻCamarosaʼ in the rates of glucose and fructose. The values of total phenolic content and antioxidant capacity were higher in soil media, and also ʻYellow Wonderʼ was found to have more total phenolic content and antioxidant capacity than ʻCamarosaʼ. In terms of mineral contents, ʻYellow Wonderʼ had higher values than ʻCamrosaʼ in both media. When the results of the study were examined in general, Camarosa red strawberry variety was found to be higher than ʻYellow Wonderʼ in pomological characteristics.

**Conclusions:**

Pomological values increased in both strawberry cultivar in soilless media. In terms of phytochemical properties, the ʻYellow Wonderʼ had higher values than the ʻCamarosaʼ. Also, Phytochemical contents were higher in the soil media compared to the soilless media.

## Background

*Fragaria vesca* (2n = 2x = 14) commonly known as the wild strawberry or alpine strawberry, is a small, diploid herbaceous perennial plant that is widely distributed throughout Europe, Asia, and North America [1]. The Yellow Wonder is a cultivar of *Fragaria vesca* that is characterized by its yellow fruit color. The mentioned plant has a low growth rate and also a herbaceous perennial plant that grows up to 15 cm. The leaves are trifoliate, with toothed edges and a bright green color. The flowers are white and five-petaled, and are followed by small, round, yellow fruit that are sweet and juicy [2]. The octoploid *Fragaria* ***×*** *ananassa* (2n = 8x = 56), commonly known as the garden cultivated strawberry, is a hybrid species (*Fragaria virginiana* x *Fragaria chiloensis*) that is believed to have originated in Europe. The plant belongs to the Rosaceae family and is widely cultivated for its edible fruit. The garden strawberry has also low growth rate and is a herbaceous perennial that grows up to 30 cm. The leaf of the plant is trifoliate and has serrated edges. The flower of the plant is white and five-petaled, and is followed by small, red, edible fruit that are sweet and juicy. The plant has a shallow root system and requires well-drained, fertile soil for optimal growth [3].

Strawberries are a popular fruit consumed worldwide due to their distinctive flavor, aroma, and attractive appearance [4]. Also they contain a wide range of phytochemicals, including flavonoids, anthocyanins, ellagic acid, and vitamin C. Flavonoids are a type of polyphenol that are found in various fruits and vegetables, including strawberries [5]. It is demonstrated that strawberries have the antioxidative and anti-inflammatory effects, which can cope against some disease including cardiovascular and cancer [6]. Anthocyanin is a type of flavonoid that give strawberries their red color [7]. Vitamin C is an important antioxidant that is found in high amounts in strawberries. It plays a key role in protecting against cellular damage and boosting the immune system. Vitamin C, which is also called as ascorbic acid, is a vitamin which is soluble in water and has your own beneficials for the human body. It has an important effect on various physiological processes, including collagen biosynthesis, immune function, and antioxidant defense. Vitamin C is not synthesized by humans and must be obtained through diet [8]. Strawberry is a fruit rich in nutrients that is full of phytochemicals and antioxidants. Minerals are essential nutrients that play various vital roles in the human body [9]. They are required for the formation of bone and tooth, regulation of the heartbeat, and the transmission of nerve impulses. Minerals also act as cofactors in different enzymatic reactions and are applied for the production of hormones and other bioactive molecules [10].

Strawberries are grown in a various locations and climates worldwide, ranging from open fields to greenhouses. The choice of the growing media is a crucial agent which has an affect on plant development, fruit performance, and quality [11, 12]. Traditionally, soil has been the most commonly used media for strawberry cultivation. However, in recent years, soilless media have gained popularity. Both soil and soilless media have their own advantages and disadvantages. Soil is a natural and readily available media that provides a stable physical and chemical environment for plant growth. However, it can also harbor soil-borne diseases and pests, which can affect on plant growth and yield. Soilless media are clean, disease-free, and can be customized to provide optimal conditions for plant growth. However, they require careful management of nutrient and pH levels, as well as regular irrigation. Different literatures have compared the application of soil and soilless media for strawberry cultivation [13, 14]. It is demonstrated that the fruit quality factors including pH, titrable acidity and also taste index (TSS/TA) are different in soil and soilless growing media of strawberries as the strawberries grown in soil had the maximum amount of pH, Ca, Zn, Fe, P, N, total soluble slides and taste index (TSS/TA) [15]. The present work was subjected to determine the effect of soil and soilless growing media on the contents of pomological and phytochemicals in two strawberries ʻYellow Gardenʼ and ʻCamarosaʼ.

## Results and discussion

**Fig. 1:**
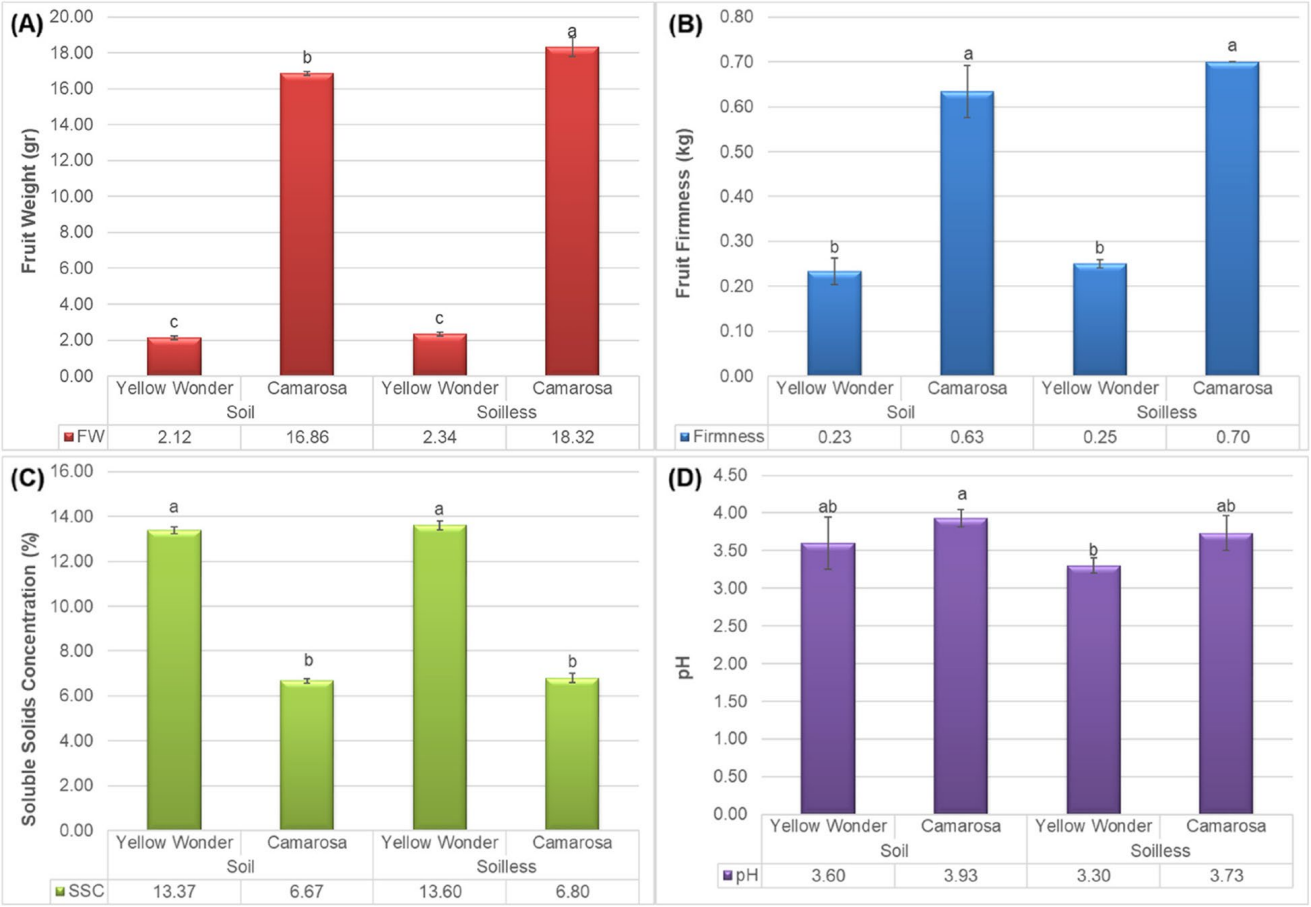
Impacts of growing media (soil vs. soilless) on the **A** fruit weight, **B** fruit firmness, **C** soluble solids concentration (SSC) and **D** pH of Yellow Wonder and Camarosa strawberry cultivars

Results of the current study showed significant impacts of growing media on the several quality attributes of Yellow Wonder and Camarosa strawberry cultivars. On the other hand, a few of the quality parameters were observed to not impacted by the growing media, especially for ʻYellow Wonderʼ. For example, growing media (soil or soilless) was noted to not significantly impact the fruit weight (Fig. 1A), fruit firmness (Fig. 1B) and soluble solids concentration (Fig. 1C) of the ʻYellow Wonderʼ fruits. However, as can be followed from the same figures, the fruit weight was significantly impacted by growing media for ʻCamarosaʼ and the soilless growing media was noted to have the highest fruit weight by 8%. The fruit firmness of the strawberries was also found to be the highest at the soilless growing media, but no significant difference was observed. Also, significant difference was noted between the Yellow Wonder and Camarosa cultivars. The fruit weight and fruit firmness was higher at the ʻCamarosaʼ, while on the other hand, Yellow Wonder cultivar was superior in terms of the fruit SSC. The fruit pH was also significantly changed among the tested cultivars and growing media (see Fig. 1D). The highest pH was noted from the ʻCamarosaʼ fruits grown in soil and was followed by ʻCamarosaʼ fruits grown in soilless and ʻYellow Wonderʼ grown in soil. The lowest pH was noted from the ʻYellow Wonderʼ fruits grown in soilless. Shortly, it was observed that the fruits grown in soilless culture has less pH than the fruits grown in soil.

According to the higher values for fruit firmness, pH, and fruit weight in ʻCamarosaʼ regardless of the growing media, it can be attributed to the genetic differences among cultivars, which is actually affected by the genotype. However, the effect of growth media and nutrition should not be overlooked, as it is clearly manifested in the fruit weight of ʻCamarosaʼ (Fig. 1A). The same findings were obtained by Ebrahimi et al. [16] who reported that the growth of strawberries on cocopeat + perlite caused to the most fruits weight. Fruit fresh weight is considered as one of the main components of plant performance in strawberries [17]. When there is no growth limiting factors such as temperature and humidity, fruit set and the fruit wight depends on the nutritional conditions of the plants and growth media during fruit set. Feeding of strawberries with appropriate nutrient solution in hydroponic cultures can caused to enhancement in weight, performance, and quality of strawberries [18]. Also, among the factors affecting on strawberry fruit weight is the ratio of nitrogen to phosphorus (N:P) and the ratio of potassium to nitrogen (K:N) where if they are close to 1 and 1.5, caused to increase the fruit weight [18]. The pH value of the berry was affected by both cultivar and growth mdia as the pH of ʻCamarosaʼ grown in the soil was more than ʻYellow Wonderʼ grown in soilless media (3.9 vs. 3.3). Increase in the antioxidants level can be one of the reasons for increase in fruit pH which is reported previously by Bordonaba and Terry [19] in strawberry. In general, the flavour of strawberry is related to the balance between fruit acidity and soluble solids concentration to get good-tasting fruits [20] which is differ according to the growing media nutrients. Furthermore, higher SSC and lower acidity concentration led to the sweeter fruits with better flavor which was observed in ʻCamarosaʼ on both soil and soilless culture compared to ʻYellow Wonderʼ (lower pH) as having more acidity is equal with an undesirable organoleptic taste [21]. Regarding to the panel test results, Treftz and Omaye [22] showed that most of the people are able to distinguish strawberries grown in soil and soilless media and interestingly 70% of participants preferred the hydroponically grown strawberry as strawberry flavor was affected by the nutrient solution used in hydroponic culture. It has been demonstrated that supplementing nutrient solution with appropriate concentration of B and Mo, caused to linearly increase in the amount of vitamin C and soluble solids concentration of strawberry [23].

The firmness of berries is the crucial factor in evaluating berry quality which influenced on postharvest quality of strawberry as the firmer ones had the longer shelf life [24]. A slight difference in fruit firmness of ʻCamarosaʼ grown in soilless culture can be related to the effect of nutrient in feeding solution compared to the grown in soil.

**Fig. 2:**
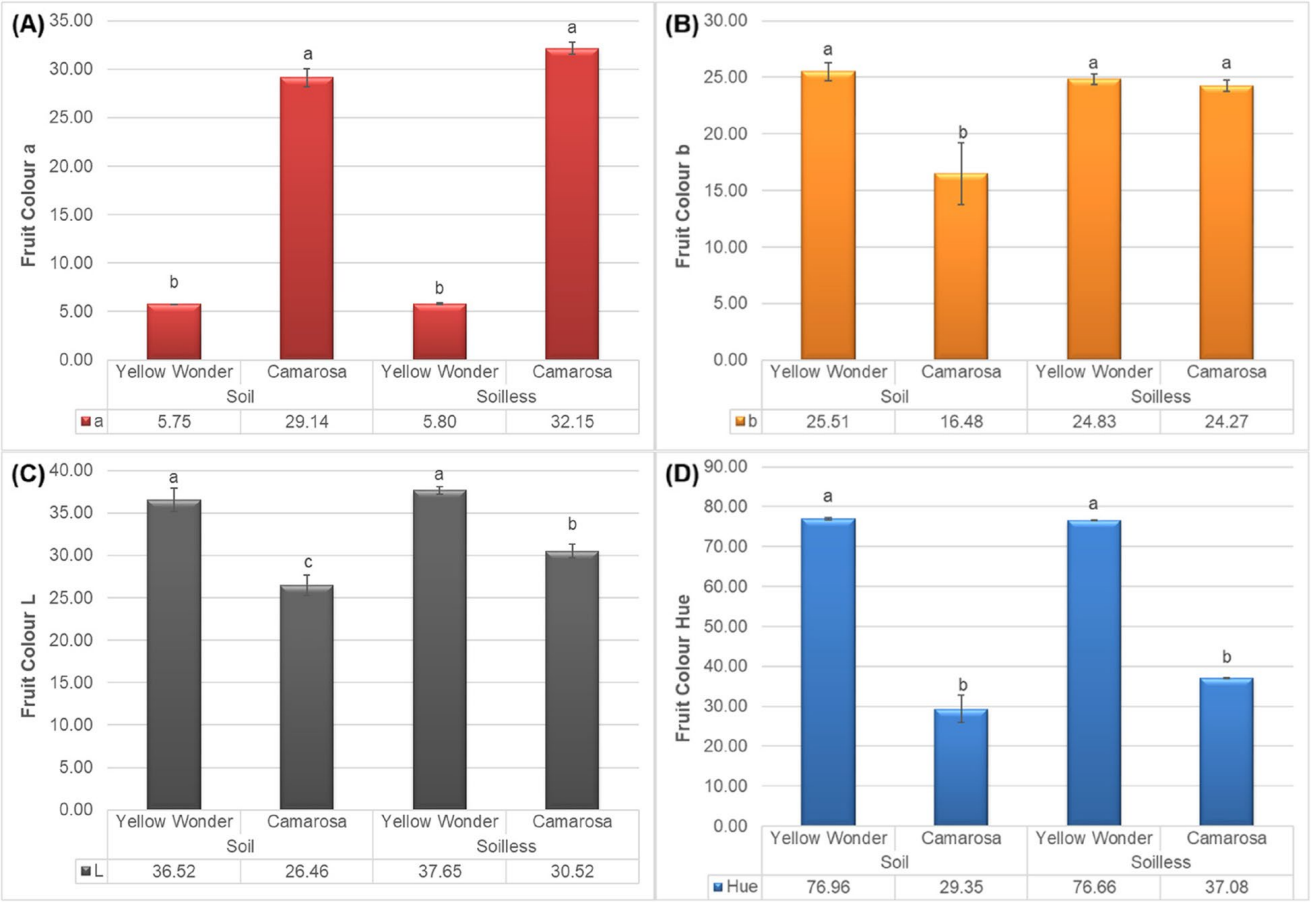
Impacts of growing media (soil vs. soilless) on the colour values **A**) a, **B**) b, **C**) L and **D**) hue of ʻYellow Wonderʼ and ʻCamarosaʼ strawberry cultivars

According to the obtained results, it can be concluded that the growing media has no significant impact on the colour a (Fig. 2A) and colour hue value on both cultivars. On the other hand, it was observed that the growing media do not impact the colour b (Fig. 2B) and colour L (Fig. 2C) of ʻYellow Wonderʼ, but significantly impact the same parameters of ʻCamarosaʼ. The colour b (Fig. 2B) value of ʻCamarosaʼ fruits increased in soilless culture, which means that the fruits grown in soilless are more yellowish than the fruits grown in soil. Similarly, the colour L (lightness) of theʻ Camarosaʼ fruits increased on fruits grown in soilless culture. Moreover, there was a significantly difference between the cultivars in terms of the colour values. In general, Camarosa cultivar was more reddish than Yellow Wonder, while the lightness of Camarosa cultivar was less than the ʻYellow Wonderʼ. Besides to that, the hue value of ʻYellow Wonderʼ was higher than the Camarosa cultivar (Fig. 2D).

The larger hue angle, can caused to a brighter berries. Color brightness is a component that lead to have an attractive and desirable fruit for retail, although the strawberries with an intense red color prefeerd by the consumers for purchase intention [25].

Our results were in line with Treftz and Omaye [22] who reported that there was no significant correlation between fruit color and juiciness for strawberries grown in the soil, although the significant correlation was observed between odor intensity and berry surface color with vitamin C. However, the effect of soilless culture on some color parameters of ʻCamarosaʼ confirmed the important role of nutrients in berry appearance in our experiment. Besides the effect of nutrient solution, light intensity also plays a key role in improving external color of strawberry [26] indicating that the external color of berry can not be estimated by nutrient or cultivar lonely.

**Fig. 3:**
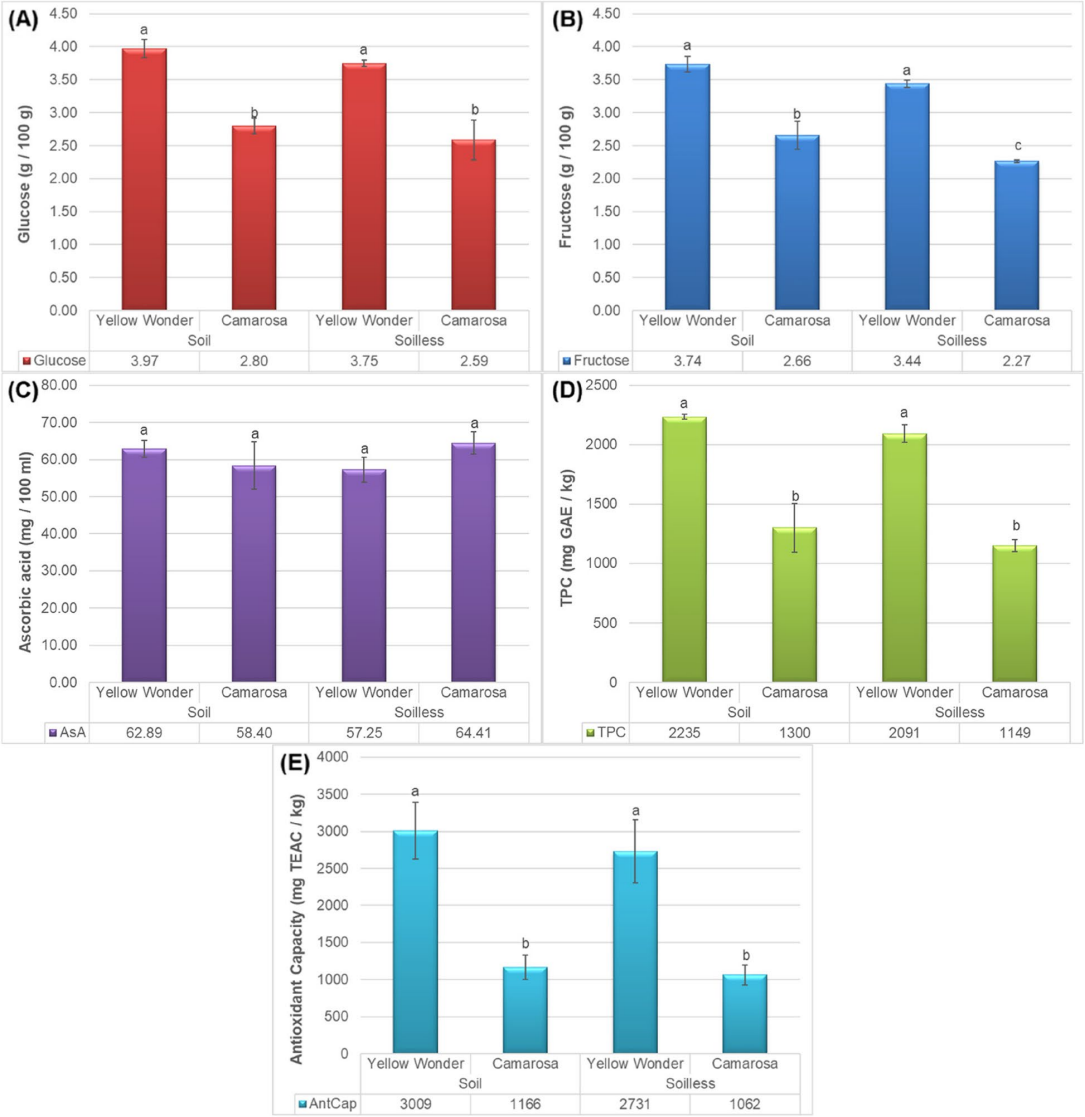
Impacts of growing media (soil vs. soilless) on the **A** glucose content, **B** fructose content, **C** ascorbic acid concentration, **D** total phenolic contents and **E** antioxidant capacity of “Yellow Wonder” and “Camarosa” strawberry cultivars

Phytochemical quality parameters of fruits are of great importance in fruit production. Results of the current work demonstrated that the growing media has slight impact on the glucose, TPC and antioxidant activity, while has no significant impact (but slight) on the ascorbic acid content and significant impact on the fructose content of the fruits (see Fig. 3). Glucose content of the tested fruits was noted to be the highest at the Yellow Wonder cultivar (3.97) which was cultivated in soil and was followed by the same cultivar grown in soil (3.75) (Fig. 3A). The different between the growing medias was not significant. The Camarosa cultivar had the lowest glucose content (mean of cultivars 2.69). Similarly, the fructose content was also higher in ʻYellow Wonderʼ and soil growing media (Fig. 3B). Contrary to the glucose content, the fructose content of the ʻCamarosaʼ fruit was found to be significantly less when cultivated in soilless media than the soil media. The amount of vitamin C in berries varied from 57.25 mg100 mL^−1^ to 64.41 mg 100 mL^−1^. The influence of different growing culturs on the amount of ascorbic acid content between cultivars was not significant as illustrated in Fig. 3C. One of the most important findings of this experiment is that the content of phenolics and antioxidant of the fruits are less at the ʻCamarosaʼ fruits while growing media has no significant impact on them (Fig. 3D). It has been proven that glucose is the most important substituting sugar in strawberrys’ anthocyanin, although other sugars inculding rutinose, arabinose, and rhamnose conjugates have been found in some strawberry cultivars [27]. Some studies indicate a relationship between glucose and anthocyanins [28]. So, the higher amount of glucose than fructose (Fig. 3A and B) was expectable in the present work because of the higher amont of TFC (Fig. 3D) and antioxidant capacity (Fig. 3E) in ʻYellow Wonderʼ compared to ʻCamarosaʼ. Our results were in agreement with Treftz and Omaye [19] who demonstrated that the amount of glucose, and fructose in strawberry and raspberry respectively were significantly higher for the soil grown berry in comparison with the soilless grown berry, although the amount of vitamin C, vitamin E and polyphenols were significantly more in soilless cultivated strawberry in comparison with the soil grown strawberry, and the amount of vitamin C and polyphenols were significantly maximum in soil cultivated raspberry in comparison with the soilless grown raspberry. The phytochemicals concentration of *F. vesca* was analyzed by Urrutia et al. [29] and 22 (poly)-phenols, such as anthocyanins, flavonols, flavan-3-ols, flavanones, hydroxycinnamic acid derivatives, and ellagic acid were recongnized in the wild strawberries (Yellow Wonder cultivar) which means that it is enriched with antioxidants. Also it was demonstrated that the plant genotype was more crucial than the environment for the production of phytochemicals. So, *F. vesca* can be considered as a potent germplasm to increase the antioxidative capacity (by genetic manipulation) of *F. × ananassa* which is more commercially planted one.

**Fig. 4:**
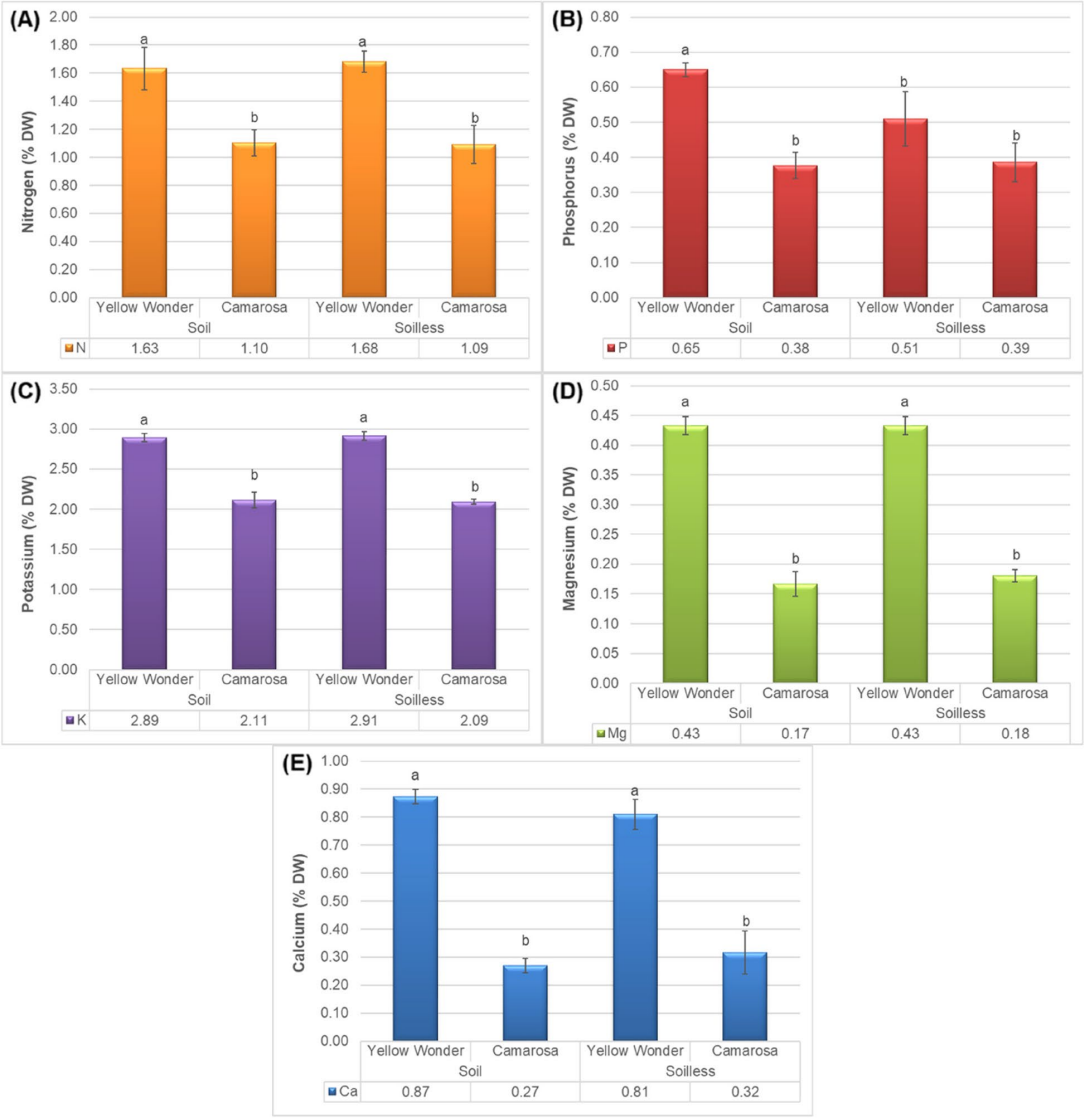
Impacts of growing media (soil vs. soilless) on the **A** Nitrogen, **B** Phosphorus, **C** Potassium, **D** Magnesium and **E** Calcium contents of “Yellow Wonder” and “Camarosa” strawberry cultivars

Nutrients, especially macronutrients are essential for plants’ growth and development. The essential macro elements including nitrogen, phosphorus, and potassium play fundamental roles in that. Nutrient deficiencies may cause stunted growth, chlorosis, death of plant tissue (even the plant), resulting in reduced plant health and crop yield. It is well-known that the roots accounts for approximately all nutrient adsorption, whereas foliar adsorption is important under some situations. Therefore, it is very important to compare the impacts of growing media on the nutrient contents of the fruits too. Results showed that the growing media had no significant impact on the nitrogen (Fig. 4A), potassium (Fig. 4C), magnesium (Fig. 4D) and calcium (Fig. 4E) content of the fruits, while significantly impacted the phosphorus content of only Yellow Wonder cultivar (Fig. 4B). However, the significant effect of cultivar was shown on nutrient pool of fruits so that all of these five nutrients are higher in Yellow Wonder cultivar and lower in ʻCamarosaʼ.

The higher amount of P in ʻYellow Wonderʼ grown in soil compared to the soilless media can be attributed to the amount of phosphorus in the nutrient solution which is limited and a determind amount in balance to other micro and macro nutrients. So, it is likely that strawberries have access to more phosphorus from the soil culture, which had unlimited sources of phosphorus, and on the other hand, due to the nature of strawberry rooting, which improves the ability to absorb less mobile elements such as P from all over the soil. The same findings were obtained by Recamales et al. [15] who demonstrated the different levels of N and P in soil and soilless cultures of strawberry.

**Fig. 5:**
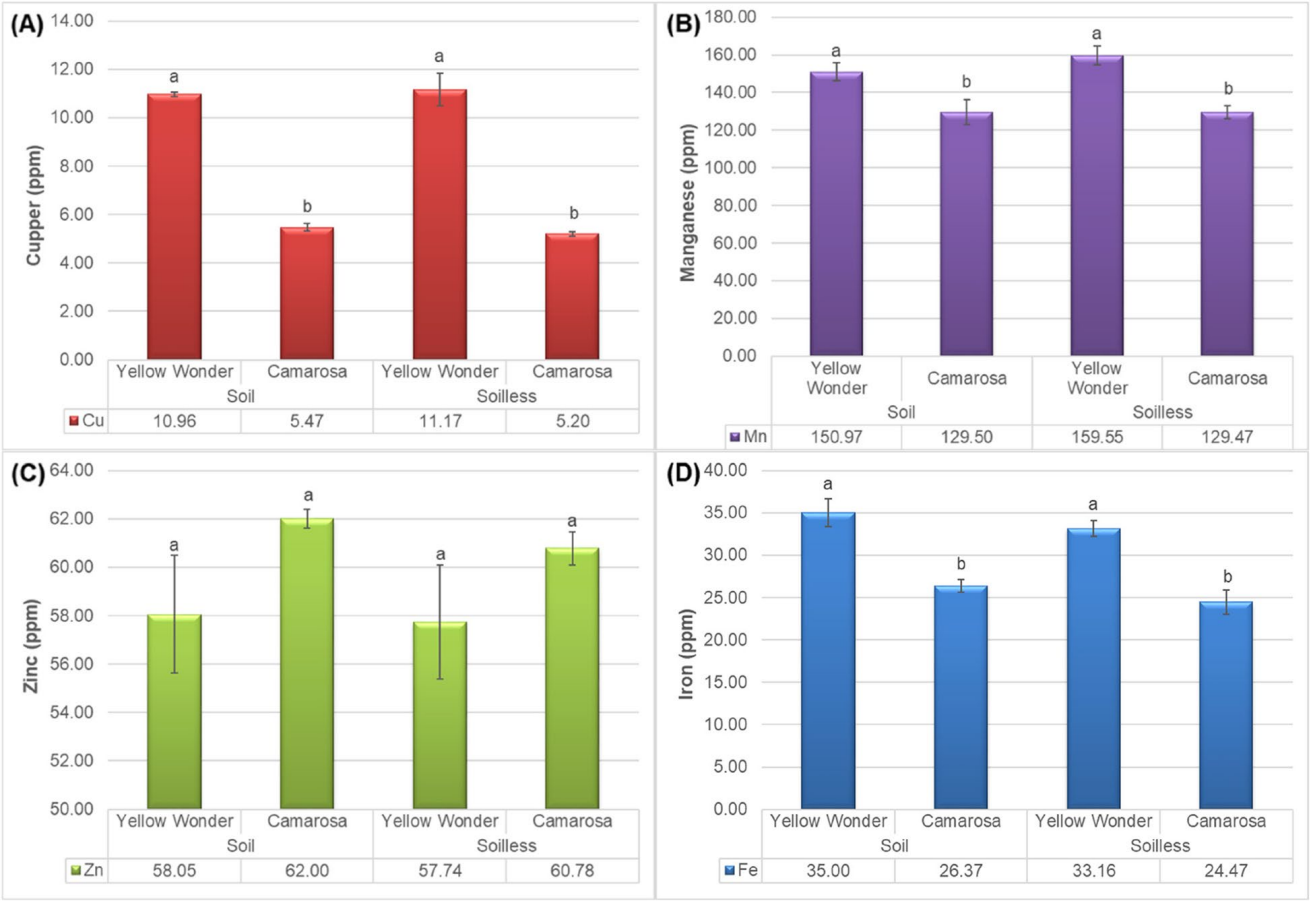
Impacts of growing media (soil vs. soilless) on the **A** Cupper, **B** Manganese, **C** Zinc and **D** Iron contents of “Yellow Wonder” and “Camarosa” strawberry cultivars

The findings about the micronutrients were found to be very similar to the results about macronutrients. Again, the Yellow Wonder cultivar was noted as superior in terms of the contents of cupper, manganese and iron (Fig. 5A and B, and D). Therefore, the zinc content of the Camarosa fruits was noted to be slightly higher than the Yellow Wonder cultivars, whereas this increase was not statistically significant (Fig. 5C). The cupper content was about double at the ʻYellow Wonderʼ as compared with ʻCamarosaʼ (Fig. 5A).

Cocopeat is an organic growing media with a good ventilation because of namerous pores in it. So, it has a high water retention capacity, high durability, high physical stability, and suitable pH [30]. Hence, it has a good potential for cations adsorption and higher cation exchange capacity (CEC). It assumed that the soilless media because of the individual properties of cocopeat (having more CEC and water retention) had the better performance in nutrient adsorption rather than soil media, although there was no difference in growing media in term of concentrations of Zi (Fig. 5C), Cu (Fig. 5A), Fe (Fig. 5D), and Mn (Fig. 5B) but the ʻYello Wonderʼ was enriched of the mentiond micronutrients in berries.

**Fig. 6:**
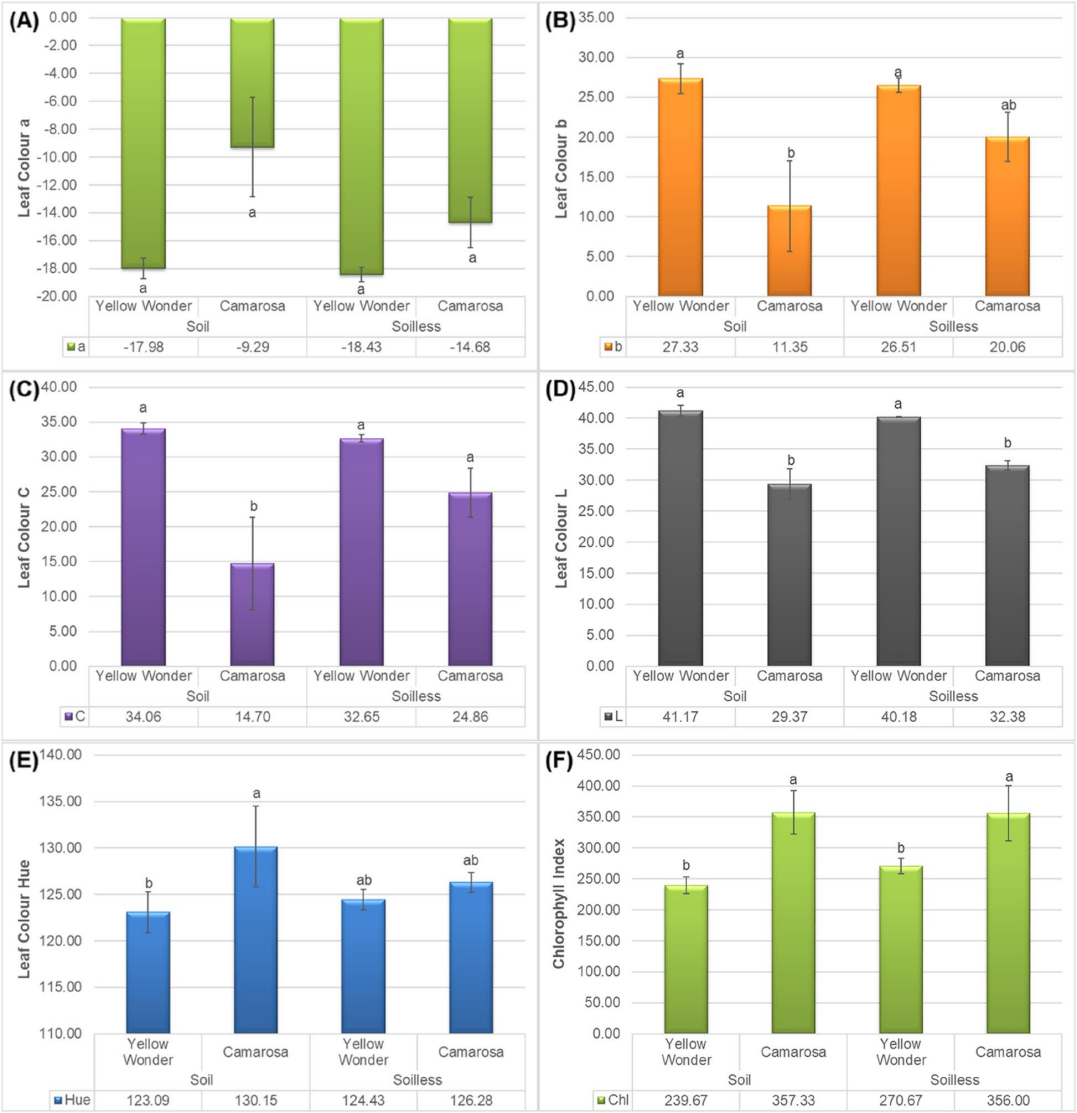
Impacts of growing media (soil vs. soilless) on the leaf colour values **A**) a, **B**) b, **C**) C, **D**) L and **E**) hue and **F**) chlorophyll index of “Yellow Wonder” and “Camarosa” strawberry cultivars

As expected, the leaf colour a value was measured as negative, which represents the green colour. No significant difference was observed among the cultivars and growing media, but the values are less at the Yellow Wonder cultivar which means greener leaves (Fig. 6A). The leaf colour b value was observed positive and significant difference was observed between the cultivars (Fig. 6B). The C (Fig. 6C) and L (Fig. 6D) values of the leaves were also observed as higher at the ʻYellow Wonderʼ and lower at the ʻCamarosa fruitsʼ. The hue value (Fig. 6E) was then found to be highest at the ʻCamarosaʼ fruits grown in soil and was followed by both cultivars grown in soilless. Another important findings of current work is that the chlorophyll index of the fruits is high at the ʻCamarosaʼ fruits and less at the ʻYellow Wonderʼ (Fig. 6F).

According to the results of Fig. 6C, ʻCamarosaʼ leaf color was the least when it was grown in soil media compared to the soilless media (Fig. 6E). Also the mentioned leaves were brighter, since they had the highest hue angle, as illustrated in Fig. 6E. These findings suggested that strawberries grown in soil may had a lighter green leaves compared to the same cultivar which is grown in soilless culture, although no significant difference was observed in leaf chlorophyll regarding to the chlorophyll readings. The higher leaf chlorophyll in ʻCamarosaʼ campared to ʻYellow Wonderʼ could be a direct effect of genotype.

**Fig. 7:**
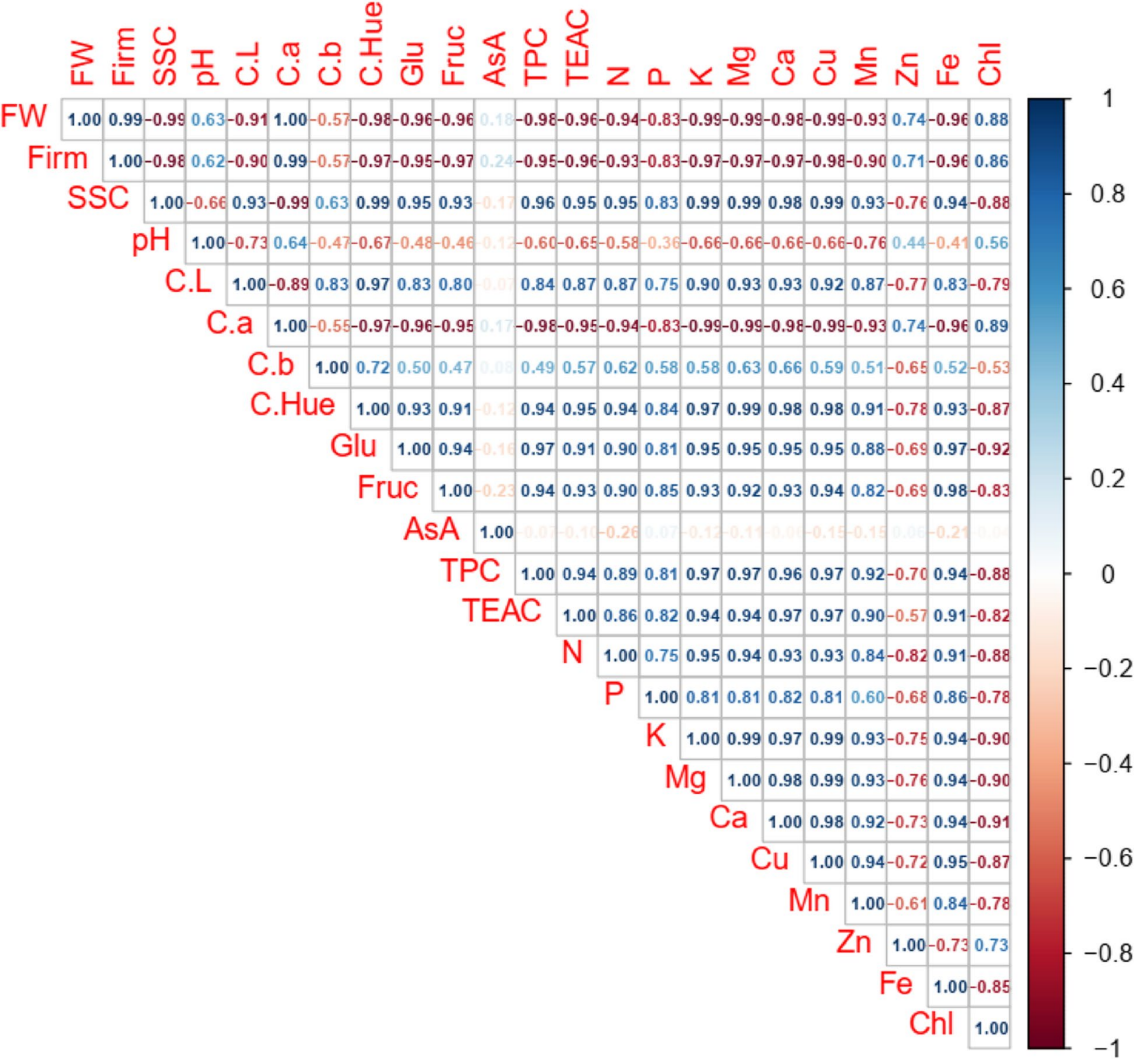
Correlations among the biochemical and fruit pomological quality characteristics of “Yellow Wonder” and “Camarosa” strawberry cultivars

Correlations analysis of current data presented very important findings for the current research (Fig. 7). For example, fruit weight was observed to have very strong positive relationship with fruit firmness (0.99), colour a value (1.00) and chlorophyll content (0.88); while it has very strong negative relationship with SSC (-0.99), colour L value (-0.91), colour hue value (-0.98), glucose content (-0.96), fructose content (-0.96), TPC (-0.98), TEAC (-0.96), nitrogen (-0.94), phosphorus (-0.83), potassium (-0.99), magnesium (-0.99), calcium (-0.98), cupper (-0.99), manganese (-0.93) and iron (-0.96). Each of the study parameters was observed to have very strong positive or negative relationships with other parameters, except for the ascorbic acid content. The relationships among ascorbic acid and others was negligible (between − 0.23 and 0.24). Details of the findings are presented in Fig. 7.

**Fig. 8:**
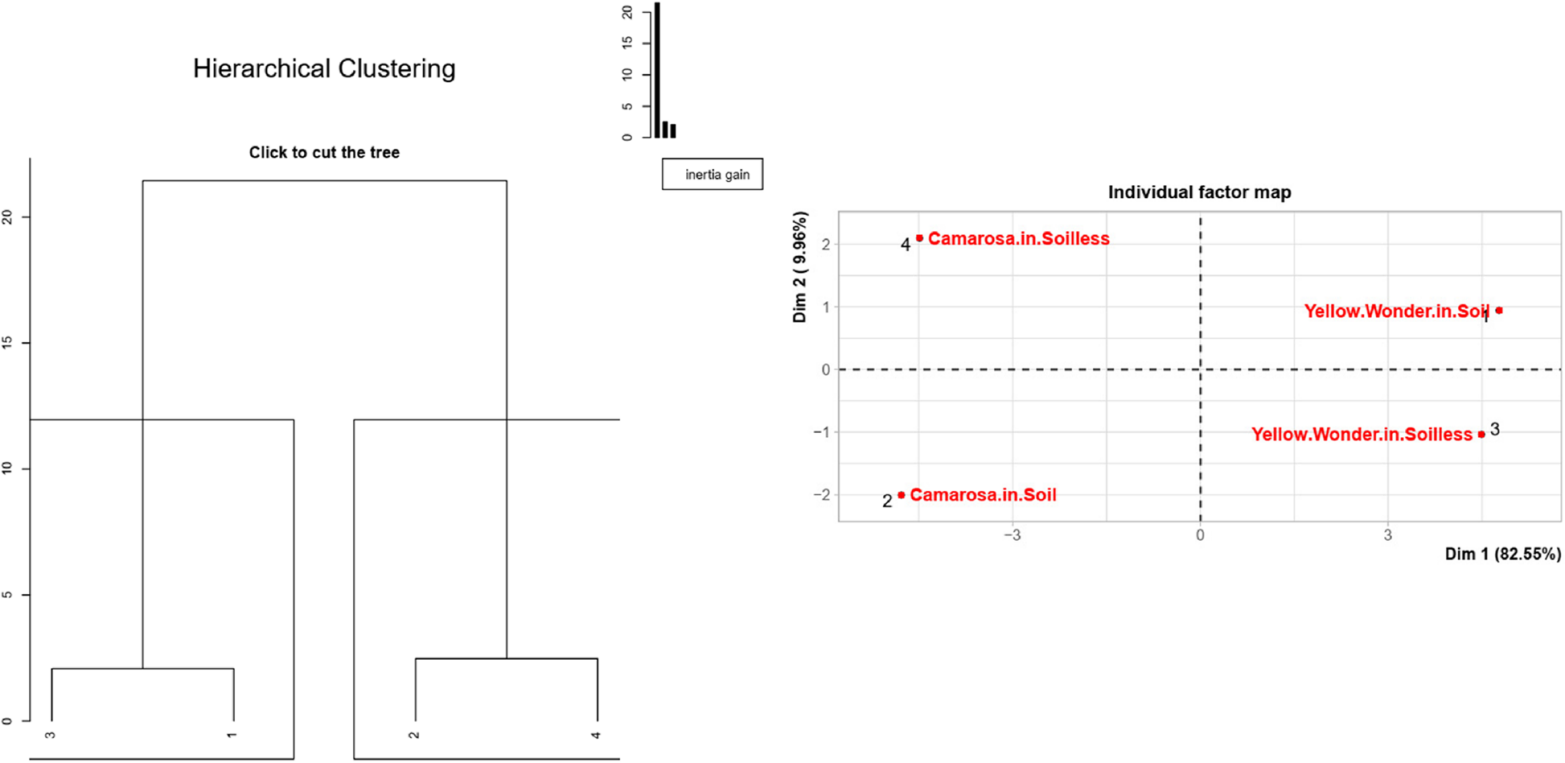
Hierarchical clustering (left) and individual factor map (right) of the “Yellow Wonder” and “Camarosa” strawberry cultivars grown in soil or soilless culture

As expected, the hierarchical clustering analysis placed the study factors into 4 district groups (see Fig. 8). This grouping clearly showed that the cultivars (Yellow Wonder and Camarosa) are significantly different from each other, while at the same time, growing conditions (soil and soilless) significantly impacts the quality parameters, and so the grouping of the factors.

**Fig. 9:**
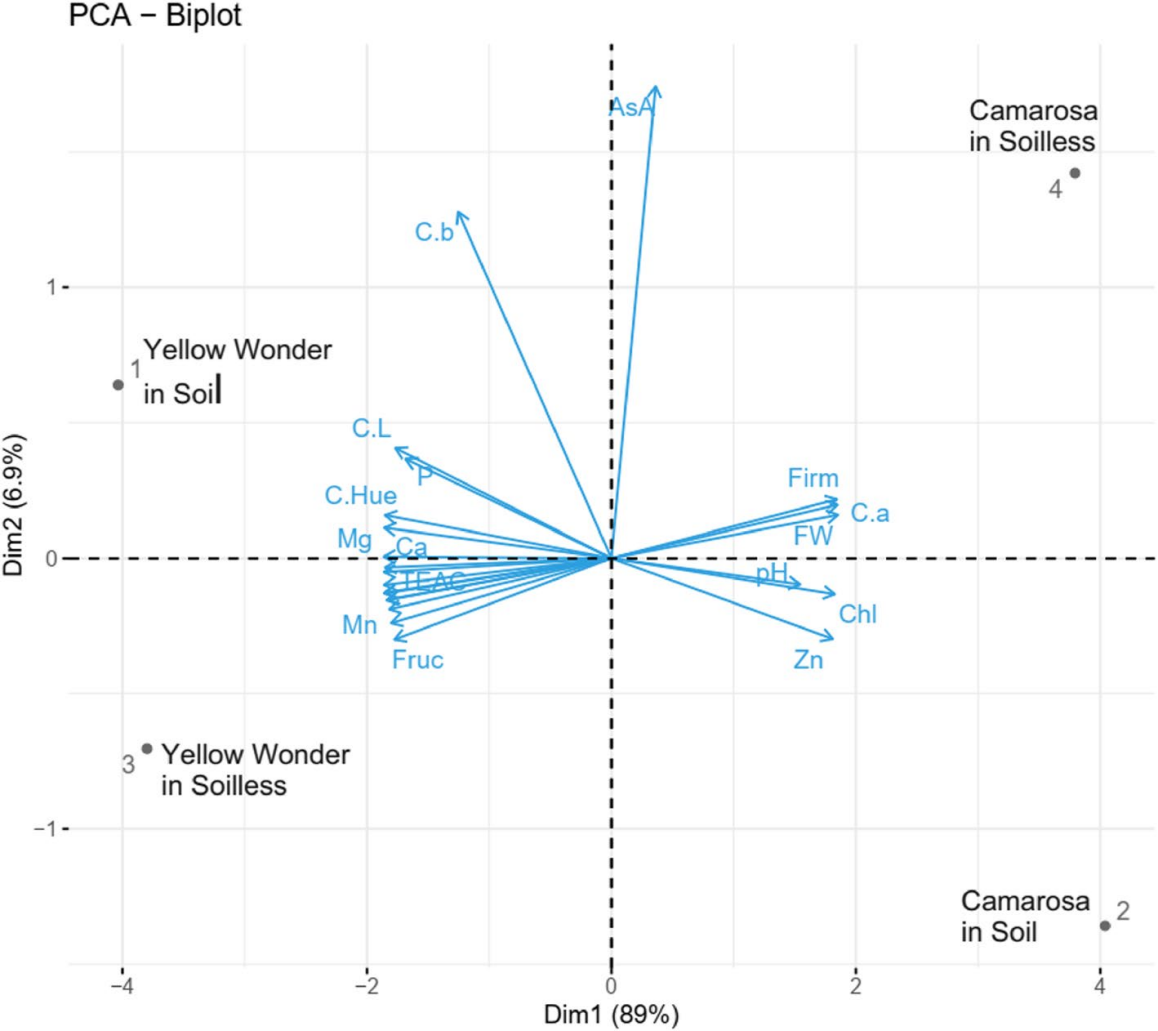
PCA-Biplot analysis of the study parameters and strawberry cultivars grown in soil or soilless culture

PCA – Biplot analysis is the best method to investigate the impacts of quality parameters on the cultivars and growing media. The Fig. 9 made it possible to make an overall evaluation of the study parameters, cultivars and growing media. It is clear from there that ascorbic acid content, fruit firmness, fruit weight and fruit colour a value are the most important parameters of ʻCamarosaʼ fruits grown in soilless culture. When the same cultivar is grown in soil, the superior parameters are pH, chlorophyll content and zinc content. On the other hand, the superior parameters of ʻYellow Wonderʼ in soil are colour b value, colour L value, phosphorus content, colour hue, magnesium content and calcium content. And finally, the superior parameters of the ʻYellow Wonderʼ in soilless growing media are glucose, fructose, TPC, antioxidant activity, nitrogen, potassium, cupper, manganese, iron and SSC.

## Conclusion

According to the qualitative traits of two cultivars of strawberry grown in soil and soilless media results, genetically differences were observed between two cultivars of strawberry, although the kind of growing media influenced on some qualitative traits of srawberry. It can be concluded that almost of sensory properties of strawberry such as berry weight and taste, and external color are influenced by the growing media while phytochemicals content of strawberry including TPC, antioxidant capacity, and reducing sugars (glucose and fructose) are likely inherited traits which is more affected by genotype than growing media. In general, regardless of people’s preference for accepting a certain cultivar, it is clear that there is a relationship between sweetness, flavor and taste with several nutrients. ʻCamarosaʼ is one of the most economically planted strawberries, although ʻYellow Wonderʼ had more TPC, antioxidant capacity, and nutrient. For this reason, it can be said that wild strawberry species that show superior biochemical content should be used in breeding and these superior properties should be transferred to commercial varieties.

## Material and method

The experiment was done in greenhouses (Soil media and Hydroponic Production System) in the Serik location of Antalya district (36° 50’- 37° 20’ N, 30° 50’- 31°10’ E; 26 m altitude), Turkey. Camarosa and Yellow Wonder strawberry cultivars were used in the experiment. ʻCamarosaʼ was obtained in 1992 by crossing *“Douglas × Cal. 85.218-605”* at the University of California. Its plants are a short day cultivar with vigorous and vertical growth [31]. ʻYellow Wonderʼ grows naturally in sunny areas on dry slopes, forest clearings and edge of forests. Voucher specimen of *F. vesca* ʻYellow Wonderʼ is deposited in the Herbarium of Akdeniz University. It is a well-known plant, valued for its nutritional as well as medicinal properties. Plant growth is medium vigorous. The most typical feature is the high aroma of fruits. Apart from fruits, also leaves and roots of wild strawberry are used as an herbal material [32]. The plant materials for wild collection was obtained under the supervision and permission of the Akdeniz University guidelines and according to national guidelines and all authors complied with all the local and national guidelines.

### Soilless media (Hydroponic Production System)

Growing bags containing cocopeat (100 cm long, 18 cm wide, 15 cm high) (pH of 5.2 to 6.8) were used as the growing media in hydroponic production, and 13 plants were planted in each bag. In the experiment, fertigation was performed by automation (INTA Crop Technology S.L, Murcia, Spain) and fertigation was planned depending on solar radiation and drainage. Nutrient solution formulations applied in the vegetative and generative period are given in Table 1. In the experiment, pH and EC settings of the nutrient solution were made during the growing season, and the pH values were kept at 5.8 and the EC value at 1.50–1.80 mS cm^−1^.

**Table 1:** Composition of the nutrient solution applied during the vegetative and generative development periods

Period of Nutrient Solution Applied	Macro Elements	Concentrations (mmol L^−1^)	Micro Elements	Concentrations (µmol L^−1^)
Vegetative Phase	NO_3_ֿ	11.5	Fe	20
	H_2_ PO_4_ֿ	1.5	Mn	20
	SO_4_^−−^	1.5	Zn	10
	NH_4_^+^	0.5	B	12
	K^+^	3.5	Cu	0.75
	Ca^++^	4.5	Mo	0.5
	Mg^++^	1.5	-	-
Reproductive Phase	NO_3_ֿ	11	Fe	20
	H_2_ PO_4_ֿ	1.5	Mn	20
	SO_4_^−−^	1.5	Zn	10
	NH_4_^+^	0	B	12
	K^+^	5.5	Cu	0.75
	Ca^++^	3.5	Mo	0.5
	Mg^++^	1.5	-	-

### Soil media

**Fig. 10:**
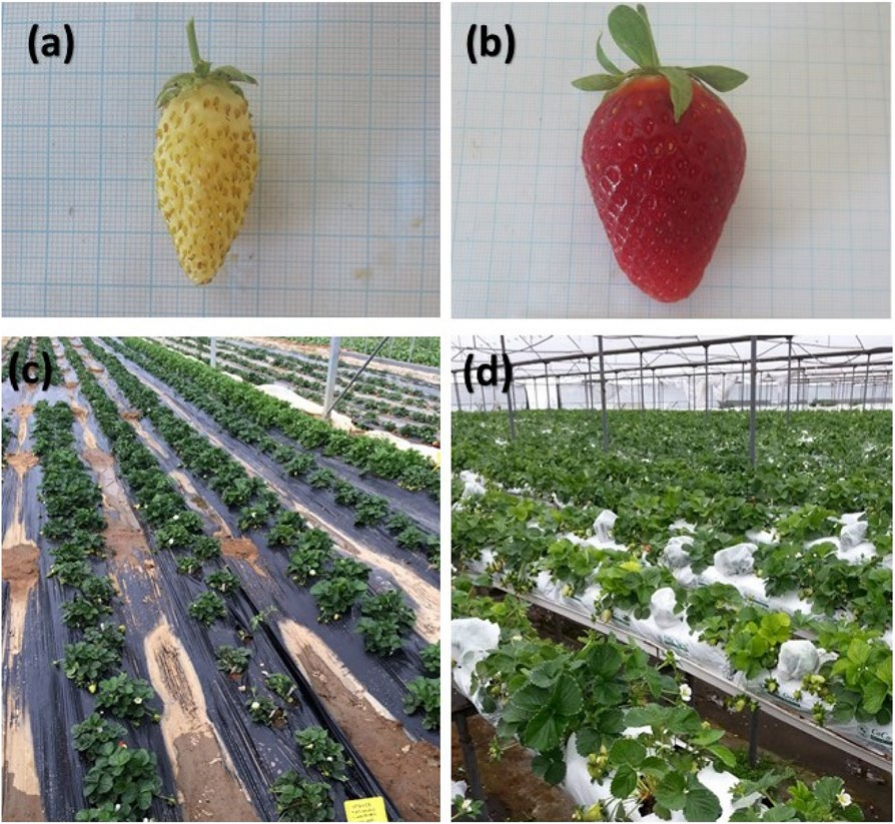
Strawberry varieties (**a** Yellow wonder; **b** Camarosa), growing media (**c** soil media; **d** soilless media) in the greenhouses

The work was done in the greenhouse with the same characteristics as the soilless production system. The soil has a heavy-clay texture, the height of the bank is 25 cm, the width of the bank is 75 cm and the walking paths are planned as 60 cm, and planting was carried out in the form of triangular planting at 30 × 30 cm distances between rows and above the rows. The drip irrigation system was used in the experiment and in the soilless production model was applied for fertigation. Soil media and Hydroponic Production System in the greenhouse had been shown in Fig. 10.

### Berry weight (g), Berry firmness (kg), total soluble solids and pH

The mature strawberries were harvested based on the harvesting indices for berry analysis. Berry weight (g), berry firmness (kg cm^−2^), soluble solids concentration and pH were examined in terms of morphological characteristics. Fruit weight (g) was measured by means of a digital precision balance (0.00 g). Firmness measurement was conducted by means of a penetrometer (FT011) with a 3 -mm probe. Firmness assay was performed on the two opposite sides of the equatorial region of 5 different berries for 1 replication, and was recorded as kg cm^−2^. Soluble solides concentration was measured with a refractometer (Model REF121, China) and recorded as ºBrix. The amount of pH was assayed using a portable pH meter (Model HI-96,100, German) at room temperature.

### Berry Colour L*, a*, b* and h°

The color of strawberries (5 berries for each replicate) was measured by means of 3NH NR20XE Precision Colorimeter (Shenzhen Threenh Technology Co., Ltd.). The surface color values of strawberries were evaluated as L* (darkness-lightness), a* (greenness-redness), b* (blueness-yellowness) and h° (hue angle).

### Total phenolic compound

Measurements of phenolics were determined regarding to the protocol of Spanos and Wrolstad [33]. In this case, 100 µL of the obtained extraction was transferred into the tubes and the lids were sealed, then 900 µL of ddH_2_O, 5 mL of Folin-Ciocalteau solution (10 times dilution in ddH_2_O) and after 3 min 4 mL of 7.5% Na_2_CO_3_ solution, were added to test tubes, respectively. This solution was vortexed for 30 s and leaved for 2 h at ambient temperature and in the dark. Then the adsorbance of the solution was recorded by spectrophotometer (Specord UV-vis L 40) at 765 nm and were expressed as mg GAE 100 g^−1^ Fw.

### Antioxidant capacity

The antioxidant capacity of strawberries were evaluated using the improved ABTS protocol [34]. The ABTS·^+^ radical cation was produced by reacting 7 mmol L^−1^ ABTS and 2.45 mmol L^−1^ potassium persulfate after 16 h incubating in the ambient temperature and dark room. The ABTS·^+^ solutions were diluted with 80% ethanol to an absorbance of 0.700 ± 0.005 at 734 nm. About 3.9 ml of ABTS·^+^ solution was added to 0.1 mL of the berry samples and vortexed completely. The solution kept at ambient temperature for 6 min and the absorbance of the solution was read at 734 nm. Various amounts (0.1, 0.2, 0.4, 0.6, 0.8, and 1.0 mmol L^−1^) of Trolox standard solution in 80% ethanol were prepared and measured under similar situations and the data were recorded as mmol Trolox 100 g^−1^ Dw.

### Ascorbic acid

In order to evaluate the amount of ascorbic acid, the berries extracts were mixed with metaphosphoric acid (6%). 5 mL of berry juice, 5 mL of acetate buffer (pH 4.0), 1 mL of 2.6 dichlorophenolindephenol and 10 mL of xylene were mixed in the falcon tubes. Then, the mixture was centrifuged at 8600 x g for 10 min at 4ºC. At the same time, a tube containing 5 mL of acetate buffer (pH 4.0), 1 mL of 2.6 dichlorophenolinindephenol and 10 mL of xylene was used as a control. The absorbance of the samples was recorded at 500 nm and the ascorbic acid content was expressed according to method described by Cemeroglu [35].

### Glucose and fructose analysis

5 g of berry pulp was mixed with ddH_2_O or meta-phosphoric acid (2.5%) reaction for each of the sugars analysis, respectively. The homogenated solutions were centrifuged at 6000 rpm for 5 min. The supernatants were purified by means of a 0.45 μm membrane filter (Iwaki Glass) before HPLC analysis, and the mobile phase solvents were degassed and were injected three times each and mean values were evaluated.

### Mineral element analysis

Measuring the nutrients were done by mixing the grounded strawberry in nitric acid and evaluated by inductively coupled argon plasma emission spectroscopy (ICAP) (Thermo Jarrell Ash ICAP 1100; Waltham, MA, USA and Spear Scientifc Digital refractometer model 300 016; Scottsdale, AZ, USA). For measuring the amount of P, K, Mg, Ca, N, Fe, Mn, Cu, and Zn a modified protocol of Wang and Lin [36]. was used.

### Leaf chlorophyll indices and colour

Leaf chlorophyll indices in strawberry leaves were determined by chlorophyll meter (FieldScoot CM1000). The color of selected strawberry leaves was measured by 3NH NR20XE Precision Colorimeter (Shenzhen Threenh Technology Co., Ltd.).

### Statistical analysis

A factorial experiment based on completely randomized design with 3 replicates was done to compare the effect of different growing media, both soil and soilless (Hydroponic Production System) culture, on the fruit quality and phytochemical contents of two cultivars of strawberry (Yellow Wonder and Camarosa) in a greenhouse. Comparison of the cultivars in different growing media was then carried with independent samples t-test at 5% significance level by using SPSS 22.0. Besides to that, several other analyses were performed by using the R 4.2.2 software and its free packages. Correlation among the quality parameters was computed and visualized by using the corrplot function. Thus, functions of res.hcpc<- HCPC(res) from the FactoMineR R package were applied to calculate and visualize cluster analysis; and function of fviz_pca_ind from the factoextra R package were applied to calculate and visualize the PCA – Biplot analysis.

## Data Availability

The datasets used and/or analysed during the current study available from the corresponding author on reasonable request.
